# Foreign Body Dactylitis

**DOI:** 10.31138/mjr.32.2.158

**Published:** 2021-05-28

**Authors:** Aggeliki Leonardou, Pantelis Kraniotis, Ioannis Koutsogiannis, Dimitrios Daoussis

**Affiliations:** 1 Physician, Private practice, Patras, Greece; 2 Department of Radiology, Patras University Hospital, Patras, Greece; 3 Department of Radiology, Olympion General Clinic, Patras, Greece; 4 Department of Rheumatology, Patras University Hospital, University of Patras Medical School, Patras, Greece

**Keywords:** Dactylitis, foreign body synovitis, MRI

**Figure 1.**
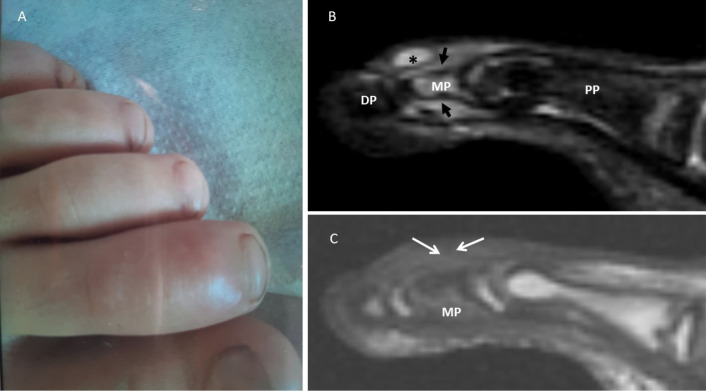
Dactylitis of the second toe (A). Sagittal STIR (B) and T1-W(C) images of the forefoot at the level of the second toe. Proximal (PP), middle (MP) and distal (DP) phalanges. The bone marrow of the MP shows abnormal signal intensity (compare with PP and DP), consisted with osteitis. There is a subcutaneous cystic lesion in the dorsal part of the MP (asterisk). Fluid is tracking along the tendons in both extensor and flexor compartments (black arrows). Just proximal to the cystic lesion a subtle (measuring 1.5mm in length), elongated, thorn-like, low signal intensity area may be appreciated (white arrows) which is perpendicular to the skin, consistent with a foreign body.

An 11-year-old boy was referred to the Rheumatology clinic with a 4-month history of dactylitis of the second toe of his left foot to rule out juvenile idiopathic arthritis. He was in excellent general status and had no symptoms, apart from relatively mild pain in his left foot while walking. The patient had no history of psoriasis, low back pain, uveitis, arthritis or enthesitis and family history for spondyloarthropathies was negative. Physical examination was unremarkable apart from swelling of his left second toe which was only mildly tender on palpation (**Figure 1A**). Interestingly, the patient recalls a history of local trauma, a few weeks prior to development of dactylitis, where he was injured by a sea urchin while he was playing in sea water. At that time, he received local treatment and a course of antibiotics. However, later on, his toe gradually became swollen and painful. He had an x-ray of his left foot which revealed only local soft tissue swelling and was treated empirically with several courses of antibiotics with no response.

An extensive laboratory workup was unre-markable with normal inflammatory markers and no autoantibodies. Taking into account the history of local trauma and the lack of an inflammatory response, which minimises the possibility of an infectious process, further imaging was ordered to seek for potential foreign bodies. An MRI scan was performed which revealed a barely perceptible foreign body in the dorsal subcutaneous tissue above the middle phalange of the second toe. A small subcutaneous fluid collection was evident just distal to the foreign body. There was also tenosynovitis of the dorsal and palmar compartment around the middle phalange (**Figures 1B** and **1C**). The foreign body was removed surgically and eventually the patient recovered fully within a few months. Black sea urchins are very common in Mediterranean waters and fortunately are not venomous. However, their spines are sharp and can easily penetrate the skin when accidently stepping on them. In the vast majority of cases, they cause only minor problems for a couple of days, but in rare cases, such as the one presented herein, sea urchin spines can infiltrate deeper tissues and cause local granulomatous reactions and synovitis. Of note, sea urchin spines are not always visible on plain x-rays and more detailed imaging may be needed.

This case is a useful reminder that dactylitis is not always a feature of inflammatory spondyloarthropathies. Whenever synovitis or dactylitis is present in a single area, a detailed history regarding local trauma should be taken. Infectious causes and presence of foreign bodies should be carefully ruled out by appropriate testing.

